# The Efficacy of Continued Sorafenib Treatment after Radiologic Confirmation of Progressive Disease in Patients with Advanced Hepatocellular Carcinoma

**DOI:** 10.1371/journal.pone.0146456

**Published:** 2016-01-08

**Authors:** Yoshiyuki Wada, Yuko Takami, Masaki Tateishi, Tomoki Ryu, Kazuhiro Mikagi, Hideki Saitsu

**Affiliations:** Department of Hepato-Biliary-Pancreatic Surgery, Clinical Research Institute, National Hospital Organization Kyushu Medical Center, Fukuoka, Japan; Taipei Veterans General Hospital, TAIWAN

## Abstract

**Background:**

Whether radiologically detected progressive disease (PD) is an accurate metric for discontinuing sorafenib treatment in patients with hepatocellular carcinoma (HCC) is unclear. We investigated the efficacy of sorafenib treatment after radiologic confirmation of PD in patients with advanced HCC.

**Methods:**

We retrospectively analyzed HCC patients treated with sorafenib at Kyushu Medical Center. Six of the 92 patients with radiologically confirmed PD were excluded because they were classified as Child-Pugh C or had an Eastern Cooperative Oncology Group (ECOG) performance status (PS) ≥3; 86 patients were ultimately enrolled.

**Results:**

Among the 86 patients, 47 continued sorafenib treatment after radiologic confirmation of PD (the continuous group), whereas 39 did not (the discontinuous group). The median survival time (MST) in the continuous group after confirmation was 12.9 months compared with 4.5 months in the discontinuous group (*p* <0.01). The time to progression in the continuous group after confirmation was 2.6 months compared with 1.4 months in the discontinuous group (*p* <0.01); it was 4.2 months and 2.1 months in patients who had received sorafenib ≥4 months and <4 months, respectively, before confirmation (*p* = 0.03). In these subgroups, the post-PD MST was 16.7 months and 9.6 months, respectively (*p* < 0.01). Independent predictors of overall survival after radiologic detection of PD were (hazard ratio, confidence interval): ECOG PS <2 (0.290, 0.107–0.880), Barcelona Clinical Liver Cancer stage B (0.146, 0.047–0.457), serum α-fetoprotein level ≥400 ng/mL (2.801, 1.355–5.691), and post-PD sorafenib administration (0.279, 0.150–0.510).

**Conclusion:**

Continuing sorafenib treatment after radiologic confirmation of PD increased survival in patients with advanced HCC. Therefore, radiologically detected PD is not a metric for discontinuation of sorafenib treatment in such patients.

## Introduction

Patients with hepatocellular carcinoma (HCC) generally have a poor prognosis. They are often diagnosed with HCC at an advanced stage or with advanced liver cirrhosis and are therefore considered unsuitable for potentially curative approaches such as resection, liver transplantation, or other locoregional therapies [1]. Sorafenib is a targeted anticancer agent that suppresses proliferation and angiogenesis by inhibiting the activities of RAF kinase and the receptors for vascular endothelial growth factor (VEGF) and platelet-derived growth factor [2]. Two large-scale, phase III clinical trials—the Sorafenib HCC Assessment Randomized Protocol trial (SHARP) study [2] and the Asia-Pacific Study [2, 3]—demonstrated that sorafenib significantly prolongs the time to progression (TTP) and improves overall survival (OS) in patients with advanced HCC. Accordingly, sorafenib is the only recognized systemic chemotherapeutic agent for patients with advanced HCC for whom resection and local therapy are not indicated [4–6]. Although it is safe and effective for patients with advanced HCC [2, 3, 7, 8], its benefits in patients with stable disease remain to be determined.

Sorafenib treatment of patients with advanced HCC is associated with early decreases in α-fetoprotein (AFP) levels [9] and neutrophil-lymphocyte ratios [10]. Early decreases in AFP levels predict good treatment efficacy [9, 11], as do increases in des-γ-carboxy prothrombin (DCP) levels [12], whereas early skin toxicity [13] and diarrhea [14] predict poor treatment efficacy. VEGF-A [15] is a positive prognostic factor for OS, and evidence suggests that early skin toxicity [16] and hypertension [17] caused by sorafenib are negative prognostic factors for OS [13]. Unfortunately, there are no biomarkers for assessing the pharmacodynamics of sorafenib [18].

Sorafenib induces a response in 2.0–3.3% of patients with stable HCC and improves OS in patients with HCC in the absence of an objective response [2, 3]. Its cytotoxicity may differ from that of cytotoxic anticancer agents that shrink tumors [19, 20]. In the SHARP and Asia-Pacific trials, sorafenib was administered until patients were diagnosed with symptomatic progressive disease (PD), and it significantly improved survival. However, whether the onset of radiologically detected PD (referred to as “radiologic PD”) is an accurate criterion for discontinuing sorafenib treatment is unclear. To address this issue, we evaluated the efficacy of continued sorafenib treatment after radiologic confirmation of PD in patients with HCC.

## Materials and Methods

### Patients

We reviewed data collected prospectively from 130 consecutive patients who received sorafenib (Nexavar; Bayer HealthCare Pharmaceuticals, West Haven, CT, USA) for treatment of advanced HCC in the Department of Hepato-Biliary-Pancreatic Surgery at the National Hospital Organization Kyushu Medical Center between July 2009 and September 2013. Of the 116 patients whose radiologic responses were assessed in accordance with the modified Response Evaluation Criteria In Solid Tumors (RECIST) [21], 92 had radiologic PD. Six of the 92 patients were excluded from the study because their liver function reserve was classified as Child-Pugh C (n = 3) or because their Eastern Cooperative Oncology Group (ECOG) performance status (PS) was ≥3 (n = 3) at the time at which PD was detected. Ultimately, 86 patients were enrolled in our investigation of the efficacy of continuous sorafenib treatment. HCC was diagnosed on the basis of the following: (i) the results of a pathological examination; (ii) a combination of specific radiologic findings obtained via dynamic multidetector-row computed tomography (MDCT) or magnetic resonance imaging (MRI); and (iii) an elevated serum AFP level according to the criteria of the American Association for the Study of Liver Diseases [22].

Sorafenib was administered to patients with advanced HCC if: (i) they were not eligible for or their disease progressed after surgery, locoregional therapy, or transcatheter arterial chemoembolization (TACE); (ii) their ECOG PS was 0–1; (iii) their liver function was classified as Child-Pugh A or B; and (iv) they had adequate hepatic function (albumin >2.5 g/dL, total bilirubin <3.0 mg/dL, and alanine aminotransferase and aspartate aminotransferase levels <5 times the upper limit of the normal range). After radiologic confirmation of PD and an explanation of their options, including continuation of sorafenib treatment, the patients selected their second-line treatments. This study was approved by the Ethics Committee of the National Hospital Organization Kyushu Medical Center and performed in compliance with the Declaration of Helsinki. All patients provided written informed consent.

### Patient Groups

Clinical features at the time of radiologic confirmation of PD were compared between two patient groups: the “continuous group” (n = 47) and the “discontinuous group” (n = 39). The continuous group continued to receive sorafenib after PD confirmation (i.e., sorafenib was the second-line treatment) at the same dose as before confirmation; no additional agents were administered along with sorafenib. The discontinuous group discontinued sorafenib treatment after PD confirmation and received the following second-line treatments: TACE (n = 11), hepatic arterial infusion chemotherapy (HAIC, n = 5), radiation (n = 3), systemic chemotherapy consisting of oral uracil and tegafur (n = 2), clinical trials (n = 4), or none (n = 14). The 16 patients who received TACE or HAIC were refractory to TACE prior to pre-PD administration of sorafenib; after pre-PD sorafenib treatment, 11 of these patients underwent TACE for new intrahepatic lesions, and 5 underwent HAIC for intrahepatic growth (n = 4) or new macrovascular invasion (n = 1). The third-line treatments (i.e., the treatments after post-PD sorafenib administration) in the continuous group were TACE (n = 11), HAIC (n = 5), radiation (n = 5), resection of lung metastasis (n = 1), and clinical trials (n = 12).

### Image Analysis of the Therapeutic Effects

Images obtained via dynamic MDCT or dynamic MRI were acquired at baseline and after 6–8 weeks of post-PD sorafenib treatment. The best radiologic tumor response was assessed in accordance with the modified RECIST [21] at 6–8 or 14–16 weeks after confirmation of radiologic PD and classified as a complete response, a partial response, stable disease (SD), or PD. We documented the cause of PD (pattern of progression): **≥**20% increase in tumor size compared with the size of a baseline lesion (intrahepatic growth or extrahepatic growth), a new intrahepatic lesion, or a new extrahepatic lesion and/or vascular invasion.

### Follow-up

All patients were followed-up at our outpatient clinic according to a standardized protocol that included tumor marker tests every 1–2 months and MDCT or MRI every 6–8 weeks until the patient’s death or last visit. At the time at which the database was locked (February 2014), the median follow-up time was 14.0 months (range, 0.7–53.9 months) and 69 patients had died; no patients were lost to follow-up.

### Statistical Analysis

Statistical analyses were performed using JMP version 11.0 software (SAS Institute, Cary, NC, USA). Categorical variables were analyzed by using the chi-square test or Fisher’s exact test, as appropriate. Continuous variables were analyzed by using Student’s *t-*test or the Mann–Whitney U test, as appropriate. TTP and OS after radiologic confirmation of PD were analyzed by using the Kaplan–Meier method, and comparisons between groups were performed by using the log-rank test. Subgroup analysis, univariate analysis, and multivariate analysis were performed by using a Cox proportional hazards model and the backward elimination procedure. A value of *p* <0.05 indicated a statistically significant difference.

## Results

### Patient Characteristics at the Time of Radiologic Confirmation of PD

The characteristics of the patients in the continuous and discontinuous groups were not significantly different (Table 1).

**Table 1:** Clinical characteristics at the time of radiologic confirmation of progressive disease.

Variable		Continuous group (n = 47)	Discontinuous group (n = 39)	*p*-value
Age		66.9	69.7	0.17
Sex	Male/female	36/11	33/6	0.35
Etiology	HBV/HCV/other	11/29/7	6/29/4	0.45
Performance status	0/1/2	15/29/3	11/25/3	0.92
Intrahepatic lesion		(n = 37)	(n = 33)	
Size (mm)	Average	29.5 ± 25.5	27.8 ± 25.5	0.78
Number	<10	17	17	0.64
	≥10	20	16	
Extrahepatic spread	(+)	22 (46.8%)	23 (59.0%)	0.26
Macrovascular invasion	(+)	8 (17.0%)	8 (21.6%)	0.68
BCLC stage	B/C	17/30	11/28	0.43
Albumin	(g/dL)	3.64 ± 0.75	3.39 ± 0.60	0.12
Total bilirubin	(mg/dL)	1.02 ± 0.48	0.94 ± 0.60	0.31
Prothrombin activity	(%)	88.0 ± 13.0	79.5 ± 14.2	0.06
Child-Pugh class	A/B	39/8	30/9	0.48
AFP	(ng/mL)	18369 ± 6360	2318 ± 6360	0.26
AFP-L3	(%)	25.5 ± 30.2	31.8 ± 26.9	0.31
DCP	(mAu/mL)	23430 ± 107097	22682 ± 45721	0.97
Initial TTP	(months)	4.1	3.4	0.35
Type of progression				
Intrahepatic growth	(+)	28 (59.6%)	28 (71.8%)	0.23
Extrahepatic growth	(+)	15 (31.9%)	16 (41.0%)	0.38
New intrahepatic lesion	(+)	17 (36.2%)	13 (33.3%)	0.78
New extrahepatic lesion	(+)	9 (19.2%)	8 (20.5%)	0.87

HBV, hepatitis B virus; HCV, hepatitis C virus; BCLC, Barcelona Clinic Liver Cancer; AFP, α-fetoprotein; AFP-L3, Lens culinaris agglutinin-reactive fraction of AFP; DCP, des-γ-carboxy prothrombin, PD, progressive disease; initial TTP, the time between initial sorafenib treatment and radiologic confirmation of progressive disease.

### Tumor Response to Continuous Sorafenib Treatment

**Fig 1:**
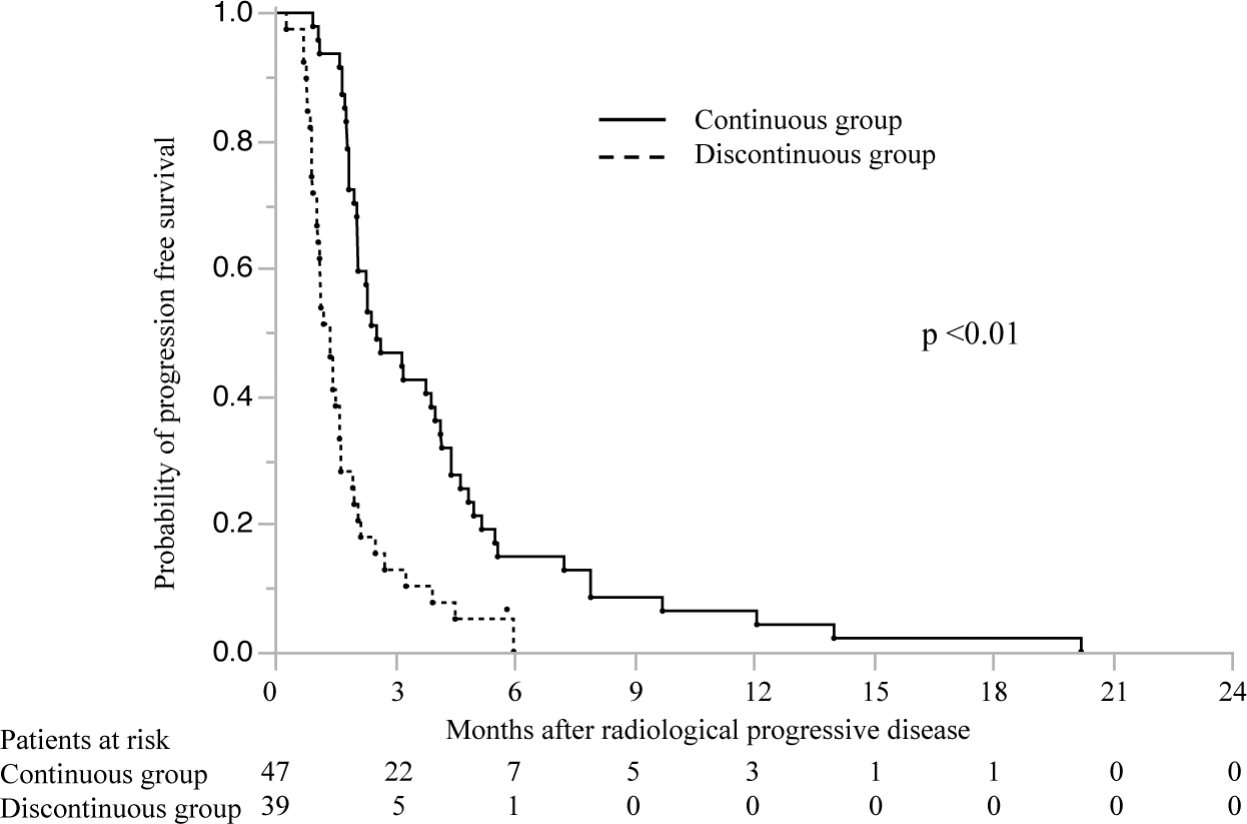
Progression-free survival in patients in the continuous (n = 47) and discontinuous (n = 39) sorafenib groups after radiologic confirmation of progressive disease. The numbers below the x-axis indicate the number of patients at risk.

All patients in the continuous group received the same dose of sorafenib after radiologic confirmation of PD as before. In this group, 5, 4, 18, 3, and 17 patients received 800, 600, 400, 300, and 200 mg of sorafenib, respectively. As assessed according to the modified RECIST[21], the best radiologic responses to continuous sorafenib treatment 6–8 or 14–16 weeks after PD confirmation were SD in 20 (42.6%) patients and PD in 27 (57.4%) patients. The median TTP was longer in the continuous group [2.6 months; 95% confidence interval (CI), 2.1–4.0] than in the discontinuous group (1.4 months; 95% CI, 1.0–1.6) (*p* <0.01) (Fig 1).

### OS in Patients with Radiologic PD

**Fig 2:**
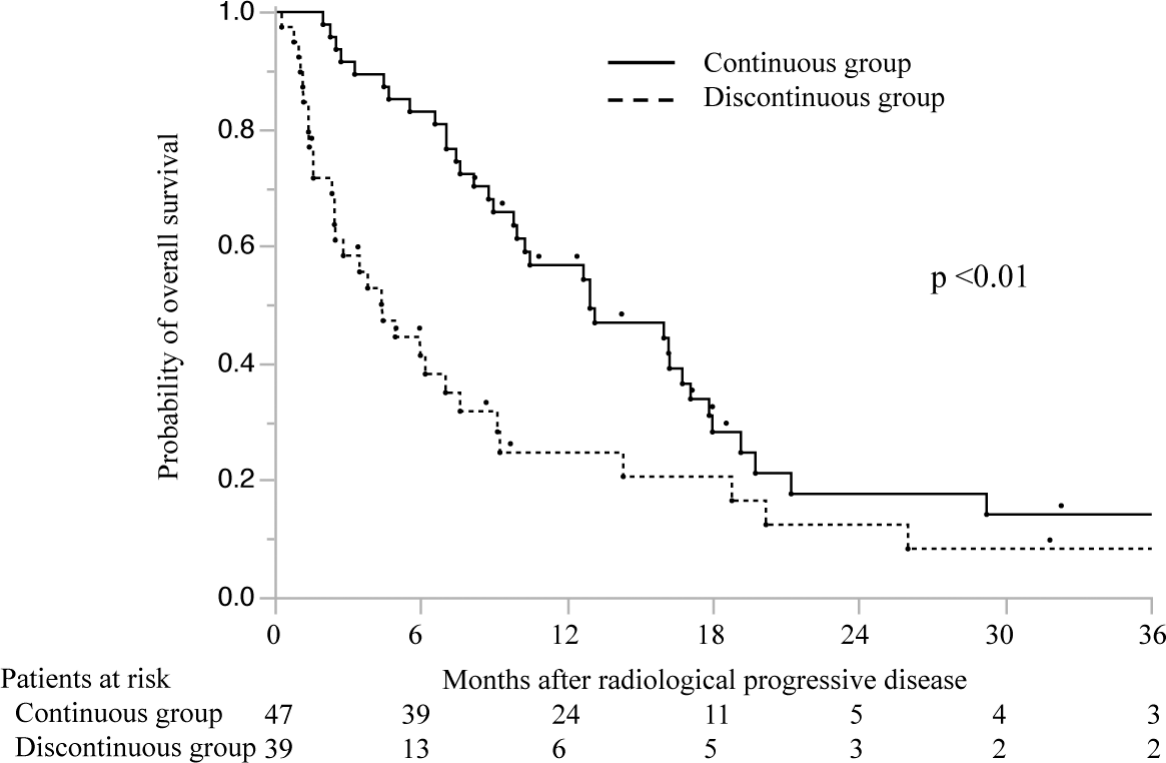
Overall survival in patients in the continuous (n = 47) and discontinuous (n = 39) sorafenib groups after confirmation of radiologic progressive disease. The numbers below the x-axis indicate the number of patients at risk.

The median survival time after radiologic confirmation of PD was longer in the continuous group (12.9 months; 95% CI, 9.8–17.1) than in the discontinuous group (4.5 months, 95% CI, 2.5–7.6, *p* <0.01, log-rank test) (Fig 2). The OS rates were 56.8% (1-year) and 17.6% (2-year) in the continuous group, and 24.7% (1-year) and 12.4% (2-year) in the discontinuous group. These results suggest that radiologic PD may not be an accurate metric for discontinuing sorafenib treatment.

**Fig 3:**
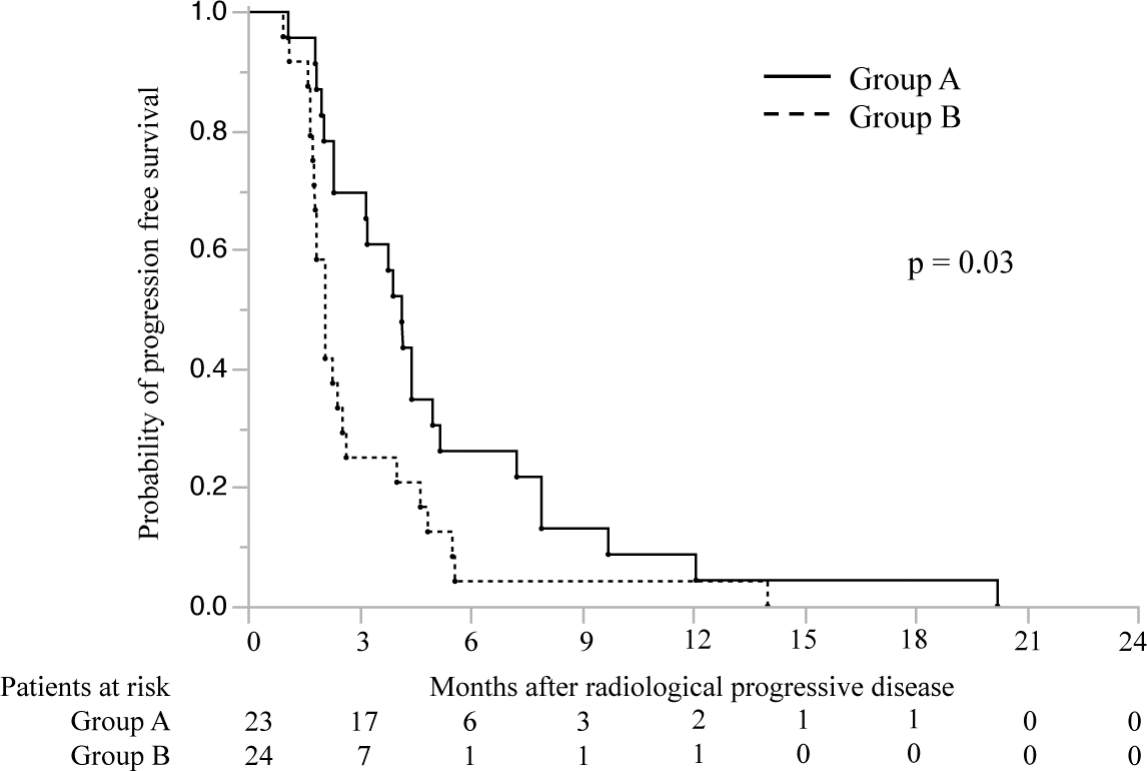
Cumulative progression-free survival rates in patients receiving continuous sorafenib treatment. The graph compares survival rates in patients who received sorafenib ≥4 months (group A, n = 23) or <4 months (group B, n = 24) before radiologic confirmation of progressive disease.

**Fig 4:**
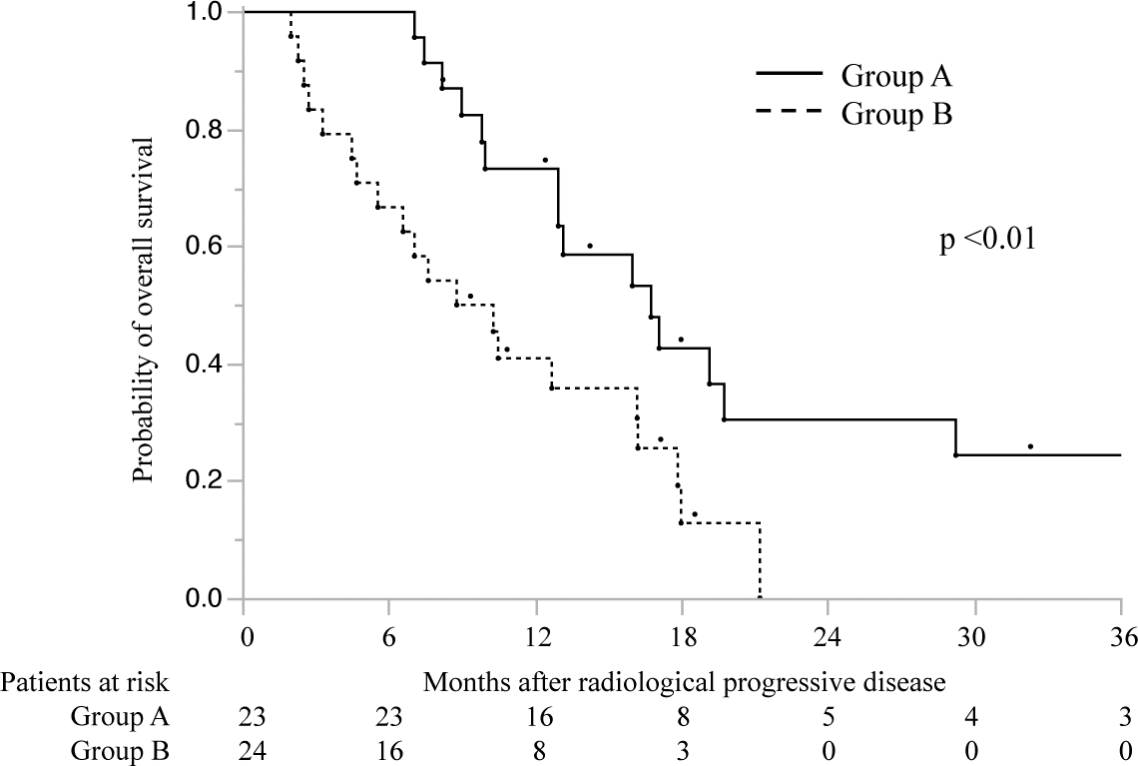
Cumulative overall survival rates in patients receiving continuous sorafenib treatment. The graph compares the survival rates in patients who received sorafenib ≥4 months (group A, n = 23) or <4 months (group B, n = 24) before radiologic confirmation of progressive disease.

Twenty-three patients in the continuous group received sorafenib ≥4 months before radiologic confirmation of PD. As assessed according to the modified RECIST[21], their best radiologic responses to post-PD sorafenib were SD (n = 15, 65.2%) and PD (n = 8, 34.8%). Twenty-four patients in the continuous group received sorafenib <4 months before confirmation, and their best radiologic responses were SD (n = 5, 20.8%) and PD (n = 19, 79.2%). Interestingly, the TTP and median survival time (MST) in patients receiving sorafenib for ≥4 months before confirmation were 4.2 months (95% CI, 1.8–2.6) and 16.7 months (95% CI, 10.0–29.3), respectively. In contrast, the TTP and MST in patients receiving sorafenib for <4 months before confirmation were 2.1 months (95% CI, 1.8–2.6) (*p* <0.01, log-rank test) and 9.6 months (95% CI, 4.7–16.2) (*p* = 0.03, log-rank test), respectively (Figs 3 and 4).

**Fig 5:**
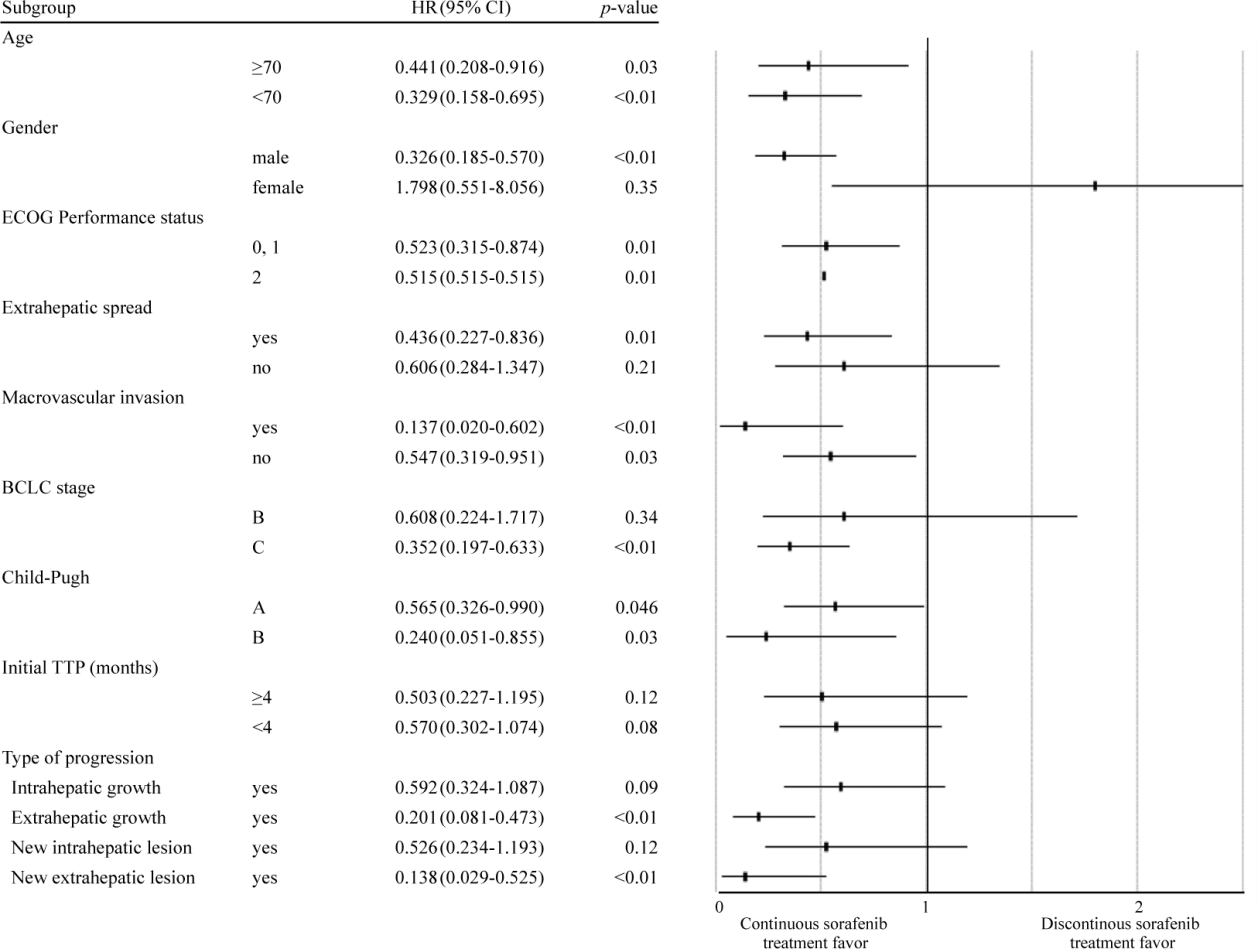
Overall survival in selected subgroups after radiologic confirmation of progressive disease according to potential prognostic factors. ECOG, Eastern Cooperative Oncology Group; BCLC, Barcelona Clinical Liver Cancer; CI, confidence interval.

A subgroup analysis showed that the survival benefits of continuous sorafenib treatment were associated with sex (male), the presence of extrahepatic spread, the presence or absence of macrovascular invasion, Barcelona Clinic Liver Cancer (BCLC) stage C, Child-Pugh status A or B, and the type of progression (extrahepatic growth or new extrahepatic lesion) (Fig 5).

### Prediction of Survival after Radiologic Confirmation of PD

Univariate analysis revealed a significant correlation between survival and the following parameters in patients with radiologic PD: age, PS, extrahepatic spread, macrovascular invasion, BCLC stage, post-PD sorafenib administration, serum AFP level, serum Lens culinaris agglutinin-reactive fraction of AFP (AFP-L3) level, serum DCP level, and type of progression (extrahepatic growth and new extrahepatic lesion) (Table 2). Furthermore, a multivariate analysis identified the following as independent predictors of OS in patients with radiologic PD (hazard ratio, 95% CI): ECOG PS <2 (0.290, 0.107–0.880), BCLC stage B (0.146, 0.047–0.457), serum AFP level ≥400 ng/mL (2.801, 1.355–5.691), and post-PD sorafenib administration (0.279, 0.150–0.510) (Table 2).

**Table 2:** Factors associated with survival in HCC patients with progressive disease.

Variable	Univariate analysis			Multivariate analysis		
	HR	(95% CI)	*p*-value	HR	(95% CI)	*p*-value
Age <70 years	0.494	(0.287–0.835)	<0.01	0.895	(0454–1.7865)	0.75
Sex (male)	1.305	(0.743–2.453)	0.37	1.630	(0.801–3.533)	0.18
Performance status <2	0.290	(0.133–0.759)	0.02	0.290	(0.107–0.880)	0.03
Extrahepatic spread	1.811	(1.117–2.972)	0.02	0.300	(0.087–1.017)	0.054
Macrovascular invasion	2.225	(1.164–4.005)	0.02	0.603	(0.216–1.659)	0.33
BCLC stage B	0.317	(0.175–0.549)	<0.001	0.146	(0.047–0.457)	<0.01
Child–Pugh class A	0.650	(0.375–1.199)	0.16	0.637	(0.325–1.300)	0.21
Sorafenib treatment post–PD	0.506	(0.312–0.826)	<0.001	0.279	(0.150–0.510)	<0.001
AFP ≥400 ng/mL	2.265	(1.286–3.899)	<0.01	2.801	(1.355–5.691)	<0.01
AFP-L3 ≥10%	1.707	(0.347–0.967)	0.04	0.944	(0.501–1.815)	0.86
DCP ≥1000 mAu/mL	2.135	(1.239–3.566)	<0.01	1.943	(0.984–3.691)	0.054
Type of progression						
Intrahepatic growth	0.791	(0.481–1.331)	0.37			
Extrahepatic growth	2.379	(1.410–3.976)	<0.01	2.064	(0.910–4.913)	0.08
New intrahepatic lesion	1.453	(0.873–2.380)	0.15	1.473	(0.820–2.603)	0.19
New extrahepatic lesion	2.386	(1.297–4.179)	<0.01	2.151	(0.997–4.513)	0.051

HR, hazard ratio; CI, confidence interval; BCLC, Barcelona Clinical Liver Cancer; AFP, α-fetoprotein; AFP-L3, Lens culinaris agglutinin-reactive fraction of AFP; DCP, des-γ-carboxy prothrombin.

*p* values were calculated using the Cox proportional hazard model.

### Safety of the Second-line Treatments in the Continuous and Discontinuous Groups after Radiologic Confirmation of PD

No patients in the continuous group experienced any serious adverse effects after radiologic confirmation of PD. The tolerability of continued sorafenib treatment after confirmation was judged acceptable. Two patients in the discontinuous group experienced serious adverse events associated with the second-line treatments (TACE, HAIC, radiation, or systemic chemotherapy). These events were refractory ascites (n = 1) and gastrointestinal tract bleeding (n = 1).

## Discussion

The purpose of the present retrospective study was to assess the usefulness of continuing sorafenib treatment after radiologic confirmation of PD in patients with advanced HCC. Its results show that post-PD sorafenib treatment lengthens survival time in a manner dependent on the timing of disease progression relative to prior sorafenib treatment.

The OS times after radiologic confirmation of PD in our study and the study by Reig et al. [23] were 9.2 months (data not shown) and 9.8 months, respectively, compared with 4.6 months in the study by Lee et al. [24]. This difference may reflect differences in the disease stage of the patients. In our study and Reig et al. [23], 52% and 33% of patients were BCLC stage B, respectively, whereas all patients in Lee et al. [24] were BCLC stage C. These findings indicate that BCLC stage C predicts poorer post-radiologic PD survival than does BCLC stage B. Moreover, Lee et al. [24] found that OS after radiologic confirmation of PD was worse when PD occurred within 4 months after initiating sorafenib treatment and suggested that second-line therapies should be administered earlier than usual in this situation. Our results also show that continuous sorafenib treatment predicts worse OS, as well as worse progression-free survival, when radiologic PD is detected <4 months after the initiation of sorafenib treatment. On the other hand, continuous sorafenib treatment may increase the survival of patients in whom radiologic PD is detected ≥4 months thereafter. Although our findings regarding effects of sorafenib administration relative to PD confirmation on survival were similar to those of Lee et al. [24], the percentage of patients with low PS and reduced liver function differed in the two studies. Sorafenib treatment is characterized by low-response, high-disease control rates, because HCCs grow slowly in most patients (2, 3). Thus, the present findings suggest that radiologic confirmation of PD is not an indication for stopping sorafenib treatment; rather, continuing treatment may provide benefits to certain patients.

Factors reported to influence OS after confirmation of PD include the pattern of disease progression, and patients with a pattern characterized by new extrahepatic lesions may be good candidates for second-line trials [23, 24]. We found that the progression pattern was not an independent prognostic indicator, although there was a trend toward significance for some patterns. Moreover, a subgroup analysis revealed that continued sorafenib treatment after radiologic confirmation of PD had a survival benefit in patients with extrahepatic growth or new extrahepatic lesions.

Conventional cytotoxic treatments are usually terminated when radiologic PD is observed, because no further benefits are expected. However, whether administration of cytostatic agents should be continued or discontinued is controversial. Treatment with sorafenib, which does not shrink the tumor, should not be based on tumor size, which is not the best indicator of efficacy [23]. Moreover, because the criteria for PD [21, 25] were determined arbitrarily without investigating the pharmacodynamics of the specific anticancer treatment, the time at which PD occurs may not necessarily predict whether sorafenib treatment will be ineffective.

The SHARP study [2] found that sorafenib treatment had significant benefits despite little tumor shrinkage and concluded that such treatment was effective. The protocol used in that study allowed patients to continue sorafenib treatment until symptomatic PD occurred after radiologic PD. On the other hand, two prospective studies [26, 27] found that continuous sorafenib treatment was not beneficial. In these studies, the dose of sorafenib in patients with radiologic PD was high (600 mg twice daily), and it is quite possible that an overdose of sorafenib caused intolerance, thus negating an accurate evaluation of outcome. Furthermore, despite randomization of the subjects in these studies, their baseline characteristics were not equivalent. Therefore, the limitations of these studies should be considered when evaluating their findings. Notably, the results of the multivariate analysis presented here support the administration of sorafenib after radiologic confirmation of PD. Although the dose of sorafenib is important [8], its effective dose after radiologic detection of PD is unknown.

The preliminary results of the Global Investigation of Therapeutic Decisions in HCC and its Treatment with Sorafenib study show that OS and TTP were longer in patients initially receiving sorafenib at 800 mg/day compared with 400 mg/day [28]. In contrast, another study found that sorafenib was equally effective at 400 mg/day and 800 mg/day [29]. In that study, a few patients tolerated an uninterrupted dose of 800 mg/day, despite experiencing a variety of adverse effects. Most patients require a decrease in the dose of sorafenib or an interruption in sorafenib treatment. The SOraFenib Italian Assessment study [30] suggested that the 50% treatment efficacy in patients receiving sorafenib at 800 mg/day might have implications for clinical practice, particularly in patients receiving tailored therapy who cannot tolerate the full dose. In the present study, the dose of sorafenib after radiologic confirmation of PD was 800 mg/day in only five (11.6%) patients and <400 mg/day in approximately 50% of patients. However, even when a low dose of sorafenib was administered after confirmation, there was a significant survival benefit.

This study has three limitations. One, the second-line treatments in the patients in the discontinuous group, which were chosen by the patients after an explanation of their options, were heterogeneous. However, radiologic responses were evaluated every 6–8 weeks after treatment administration, and patients with severe deterioration in PS and liver function were excluded from the study. Two, the study design was retrospective and non-randomized. Three, the size of the study cohort was small. To confirm the efficacy of continued sorafenib treatment after radiologic detection of PD, further prospective studies with a larger number of subjects are required. There is no useful second-line treatment after sorafenib, and the present results suggest that continuing sorafenib treatment after radiologic confirmation of PD prolongs the survival of patients with advanced HCC. These findings are consistent with those of the pivotal SHARP study [2].

## Conclusion

This study demonstrates the efficacy of continued sorafenib treatment after radiologic confirmation of PD in patients with advanced HCC. Therefore, radiologic PD is not a metric for discontinuation of sorafenib treatment in such patients.
